# Effectiveness of Robot-Assisted Upper Extremity Function Training (Gloreha) on Upper Extremities Function After Stroke: Systematic Review

**DOI:** 10.2196/68268

**Published:** 2025-06-05

**Authors:** Chirathip Thawisuk, Sopida Apichai, Waranya Chingchit, Jananya P Dhippayom, Teerapon Dhippayom

**Affiliations:** 1 Department of Occupational Therapy, Faculty of Associated Medical Sciences, Chiang Mai University, 110 Intawaroros Rd., Sripoom, Chiang Mai, 50200, Thailand, 66 53-949-259; 2 The Research Unit of Evidence Synthesis (TRUES), Faculty of Pharmaceutical Sciences, Naresuan University, Phitsanulok, Thailand; 3 Department of Pharmacotherapy, University of Utah College of Pharmacy, Salt Lake City, United States

**Keywords:** rehabilitation, robot-assisted therapy, stroke, systematic review, upper-limb, PRISMA, Preferred Reporting Items for Systematic Reviews and Meta-Analyses

## Abstract

**Background:**

The Gloreha (Idrogenet SRL) is a robotic device that enhances conventional rehabilitation for improving upper extremity function after stroke, but comprehensive evidence on its effectiveness is still lacking.

**Objective:**

The objective of this study was to evaluate the effectiveness of the Gloreha device on upper extremity function and activities of daily living (ADLs) in patients with stroke.

**Methods:**

PubMed, Cochrane CENTRAL, CINAHL, Embase, and EBSCO Open Dissertations were searched from January 2013 to January 2024. The inclusion criteria were randomized controlled trials involving adult patients with stroke that compared rehabilitation with the Gloreha device to conventional rehabilitation and reported upper extremity function or ADLs outcomes. All included studies underwent bias risk assessment using the Revised Cochrane risk-of-bias tool for randomized trials.

**Results:**

Out of 1123 studies identified, 3 randomized controlled trials involving 83 participants were included. Of these, 2 trials combined Gloreha training with conventional rehabilitation, while in another trial, patients engaged solely in the training by the Gloreha device. The Gloreha, whether integrated with conventional rehabilitation or used independently, has the potential to enhance motor function and functional ability in survivors of stroke.

**Conclusions:**

Gloreha passive training with conventional rehabilitation improves upper extremity function post stroke, but ADL effects and long-term optimal dosing require further research.

## Introduction

Stroke is the second leading cause of death and the third leading cause of disability worldwide [1,2]. In 2019, there were 12.2 million new cases of stroke, and 101 million people were living with stroke-related conditions [2]. Furthermore, the rate of new strokes appears to be increasing [3]. Studies have shown that up to 50% of the survivors of stroke experience more than 6 impairments [4,5], with upper extremity dysfunction being particularly debilitating [6-9]. Upper extremity dysfunction limits the ability of the survivors of stroke to perform daily activities independently [10-12]. Despite efforts to improve motor function through conventional rehabilitation approaches, achieving optimal recovery outcomes remains a challenge [13].

Various interventions exist to improve upper extremity function after stroke, including bilateral arm training, constraint-induced movement therapy, robotics, strength training, task-specific training, virtual reality, and mirror therapy [14]. In recent years, robot-assisted therapy (RAT) has emerged as an innovative technology to augment conventional rehabilitation [15]. RAT offers high-repetition or high-intensity practice, which may induce neuroplasticity in patients with stroke [16]. Recent evidence suggests RAT is more effective than traditional therapy approaches for stroke rehabilitation [17]. Robotic devices have shown a moderate positive effect on improving hand and arm function in patients with stroke, with effects on activities of daily living (ADLs) and upper extremity function comparable with conventional therapy (CT) [17]. Although mechanically guiding patients’ hands through grasping tasks is a relatively novel approach, various commercially available robotic devices have been developed [18]. One such device is the Gloreha (Idrogenet SRL), a robotic glove that allows customized therapy sessions focused on specific tasks. It consists of a glove and monitor providing visual feedback, with its key innovation being precise control of sequential finger movements to enhance the learning process [19]. Gloreha was selected for this review due to its unique ability to provide precise, individualized, finger-by-finger training and integrated visual feedback, features that are particularly important for restoring fine motor skills in survivors of stroke [19].

Despite growing interest in its clinical application, comprehensive evidence regarding the effectiveness of Gloreha, especially its impact on upper extremity function and ADLs after stroke, remains limited. Although 2 systematic reviews and meta-analyses suggest that RAT, including Gloreha, can enhance upper extremity function post stroke [20,21], further investigation is needed. These reviews evaluated various robotic devices, including exoskeletons and end-effector machines, which may activate different brain pathways during the recovery process. For example, devices such as AMADEO (Tyromotion Inc) and Hand of Hope (Rehab-Robotics Company Ltd) are generally designed to facilitate finger movement in a static arm position with a linkage system, whereas Gloreha facilitates finger movement in a dynamic arm position through a cable-driven mechanism [22]. However, they did not provide specific insights into the influence of Gloreha on upper extremity function and ADLs. Furthermore, although Gloreha has potential applications in other populations with upper-limb disabilities, the high prevalence of fine motor impairments in survivors of stroke and the consequent impact on daily living justify focusing this review exclusively on the population with stroke. Comprehensive evidence on the effects of Gloreha device would equip health care professionals with the reliable information needed to deliver personalized, high-quality rehabilitation interventions for patients with stroke. Thus, this research aims to systematically collect evidence from the literature and summarize the effects of robot-assisted training on upper extremity function and ADLs in patients post stroke, with a specific focus on the Gloreha device.

## Methods

### Ethical Considerations

This systematic review was reported according to the PRISMA (Preferred Reporting Items for Systematic Reviews and Meta-Analyses) statement [23] (Checklist 1). The study protocol received exempt approval from the Ethics Committee, Faculty of Associated Medical Sciences, Chiang Mai University.

### Data Source

Randomized controlled trials (RCTs) were identified by searching the following databases: PubMed, the Cochrane Central Register of Controlled Trials, CINAHL, Embase, and EBSCO Open Dissertations from January 2013 to January 2024. Our research focused on the effects of robot-assisted upper extremity function training, a field that has advanced rapidly in recent years. To ensure that the included evidence reflects current robotic technologies, treatment protocols, and clinical applications, we limited our search to studies published between 2013 and 2024. This 10-year time frame was selected based on evidence suggesting that restricting the search to the past decade yields credible and timely results [24]. Studies published before 2013 were excluded, as they may involve outdated technologies and methodologies, limiting their relevance to modern rehabilitation practices. The following keywords were searched: stroke, Gloreha, robotics, and upper extremity. (Refer to Multimedia Appendix 1 for a comprehensive list) Search strategies were developed for each database using both free-text terms and the controlled vocabulary. The reference lists and tracked citations of included studies were searched to identify additional trials through the Scopus database. Scopus was included in our search strategy to extend coverage beyond primary health care databases by enabling reference list screening and citation tracking. Its broad disciplinary scope and built-in citation analysis tools allowed us to identify additional relevant studies that are not indexed in primary databases, recommended in the Cochrane Handbook, ensuring a more comprehensive literature search.

### Study Selection

Records were screened through a systematic review application, Catchii [25]. Duplicate removal was performed using Catchii’s duplicate detection method, and the results were manually verified before removal. The selection process occurred in 2 stages. First, the titles and abstracts of identified studies were screened, followed by full-text screening. Study inclusion was decided independently by 2 reviewers (CT and SA) based on the following inclusion criteria: (1) the studies were RCTs, (2) the participants were adult patients (≥18 y) affected by ischemic or hemorrhagic stroke, (3) the rehabilitation involved Gloreha robotic device compared with other rehabilitation methods (conventional occupational therapy or physiotherapy or both), and (4) the studies included upper extremity function or ADLs outcomes, assessed by validated measures. Disagreements were resolved through discussion between the 2 reviewers or with a third reviewer (JPD) when consensus could not be reached.

### Data Extraction

Two reviewers (CT and SA) independently extracted data from each included trial using a predefined data extraction form. Disagreements were resolved through discussion or, if necessary, adjudication by a third reviewer (JPD). The following variables were extracted: title, authors, year of publication, journal of publication, participants (number, mean age, and gender), study design, rehabilitative intervention details (frequency and duration of sessions, and Gloreha parameters such as passive mode and active assistive mode), conventional rehabilitation intervention details (frequency and duration of sessions, rehabilitation techniques, and provider), outcome measures, results, and conflict of interest of each trial.

### Assessment of Methodological Quality

The methodological quality of the included trials was measured using the Revised Cochrane risk-of-bias tool for randomized trials (RoB 2) [26]. The RoB 2 scale includes 5 bias domains (bias arising from the randomization process, bias due to deviations from intended interventions, bias due to missing outcome data, bias in measurement of the outcome, and bias in selection of the reported result). The overall risk of bias for each trial was determined as low, high, or some concerns.

## Results

### Study Selection

**Figure 1.**
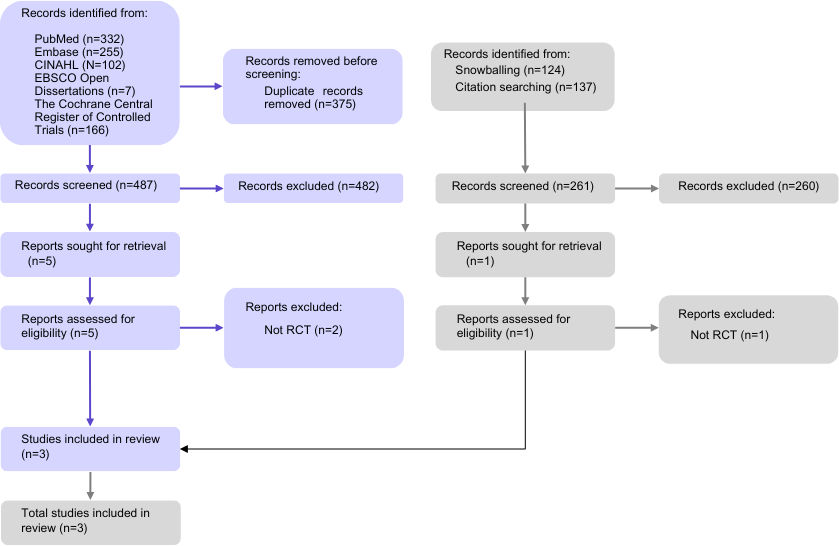
PRISMA (Preferred Reporting Items for Systematic Reviews and Meta-Analyses) diagram of study selection. RCT: randomized controlled trial.

The primary search identified 862 references, as illustrated in Figure 1. After removing duplicates, 487 references were screened, and 482 were excluded because they did not meet our criteria. Furthermore, 5 full-text publications were reviewed, and finally, 3 met the inclusion criteria. An additional search was conducted on the reference lists and citation tracking, yielding 261 references. After screening, only 1 reference was reviewed as a full text, but it did not meet the inclusion criteria since the design was not an RCT (refer to Multimedia Appendix 2 for a list of excluded studies). In total, 3 studies were included.

### Study Characteristics

The study characteristics of the 3 included studies are summarized in Table 1. Furthermore, 1 study used randomized cross-over trial design [27] and 2 studies had a parallel randomized controlled design [28,29], and were conducted in Italy [28,29] and Taiwan [27].

**Table 1.** General characteristics of the 3 included studies.

Study	Country	Study design	Sample size	Characteristics of participants, n (%) or mean (SD)						Reported COI^a^
				Age	Sex	Etiology	Affected side	Baseline-motor performance	Poststroke duration (d)	
Lee et al [27]	Taiwan	RCT^b^ with crossover	N=24 RAT^c^=14 CT^d^=10	RAT: mean 59.56 (SD 8.29) CT: mean 53.50 (SD 12.33)	RAT: male=9, female=5 CT: male=7, female=3	RAT: infarction=9 (64%), hemorrhage=5 (36%) CT: infarction=4 (40%), hemorrhage=6 (60%)	RAT: left=9, right=5 CT: left=8, right=2	MAS^e^ RAT:proximal mean 1.36 (SD 0.75), distal mean 1.29 (SD 0.91) CT: proximal mean 1.50 (SD 0.53), distal mean 1.80 (SD 0.63) Brunnstrom stage RAT: proximal mean 3.36 (SD 1.15), distal mean 3.29 (SD 1.20) CT: proximal mean 3.00 (SD 0.82), distal mean 2.90 (SD 0.8)	RAT: mean 882.00 (SD 957.67) CT: mean 883.30 (SD 1020.49)	Not reported
Vanoglio et al [28]	Italy	Pilot RCT	N=27 RAT=14 CT=13	RAT: mean 72 (SD 11) CT: mean 73 (SD 14)	RAT: male=7, female=7 CT: male=7, female=6	RAT: infarction=12 (75%), hemorrhage=4 (25%) CT: infarction=12 (75%), hemorrhage=4 (25%)	RAT: left=11, right=4 CT: left=10, right=5	RAT: mean 15.2 (SD 6.8) CT: mean 17.8 (SD 7.9)	None declared	
Villafañe et al [29]	Italy	Double-blind RCT	N=32 RAT=16 CT=16	RAT: mean 67 (SD 11) CT: mean 70 (SD 12)	RAT: male=11, female=5 CT: male=10, female=6	RAT: infarction=10 (67%), hemorrhage=5 (33%) CT: infarction=9 (60%), hemorrhage=6 (40%)	RAT: left=9, right=7 CT: left=8, right=8	RAT<90^f^ CT<90^f^	None declared	

a COI: conflict of interest.

b RCT: randomized controlled trial.

c RAT: robot-assisted therapy.

d CT: conventional therapy.

e MAS: Modified Ashworth Scale.

f All participants had the onset not more than 3 months.

### Participants

The total number of participants from all the included trials was 83, with mean ages ranging from 53.5 to 73 years old. All trials included both male and female participants. Regarding etiology, most participants had experienced ischemic strokes, while a smaller percentage had hemorrhagic strokes. The studies differed in the timing of therapy initiation after stroke. Furthermore, 2 studies focused on the acute to subacute phase (less than 3 mo post stroke) [28,29], while 1 study included participants in the chronic phase (more than 6 mo post stroke) [27].

### Intervention

In 2 trials, training with the Gloreha was combined with conventional rehabilitation programs and compared with conventional rehabilitation programs alone [28,29]. In another trial, patients engaged solely in upper extremity training with the Gloreha device, which was also compared with conventional rehabilitation programs [27]. The passive mode of the Gloreha device was consistently employed across the studies [27-29], in which participants’ fingers were guided through various movements, such as finger counting, making a fist, pinching, and synchronous finger movements. In 1 study, the Gloreha device was used in an active-assisted activities mode combined with game mode and task-oriented activities, such as grasping a box [27]. On average, treatment lasted 3‐6 weeks, with each session lasting 30‐60 minutes per day, and a frequency of 2‐5 times per week. Details of the interventions among included trials are summarized in Table 2.

**Table 2.** Intervention details of the 3 included studies.

Study	Characteristics of intervention					
	Intervention	Comparator	Duration (wk) and duration per each session (min)	Duration of therapy (wk)	Follow-up	Outcome
Lee et al [27]	60 minutes session of robot-assisted therapy which included 20 min warm-up, 10 min passive ROM^a^ with Gloreha, 30 min active-assisted activities (eg, task-oriented bimanual activities active-assisted activities and game)	60 minutes of conventional therapy which included 20 min warm-up and 40 min conventional OT^b^ program (eg, task-oriented bilateral hand, grasp-and-release, and pinch activities)	2 days a week, 60 min	6 weeks (1 month washout period)	Baseline and postintervention	Upper extremity function FMA-UE^c^ BBT^d^ Grip strength Sensory function Sensory test ADL^e^ MBI^f^
Vanoglio et al [28]	Standard rehabilitation (PT^g^/OT) 6 days per week plus 40-min robotics rehabilitation session, 5 days/week. In this program, the affected hand was passively moved by the Gloreha. Each training session consisted of 6 parts (7 min for each part): 17 cycles of digital joint flexion and extension, 23 cycles of movements (counting from 1 to 5), 70 cycles of thumb-finger opposition, 28 cycles of wave-like finger movement, 42 cycles of fist opening and closing, and 20 cycles of flexion and extension of the finger alternated with flexion and extension of the thumb.	Standard rehabilitation (PT/OT) 6 days per week plus 40-min hand rehabilitation session, 5 days/week. In this program, the affected hand was passively moved by PT. The activities were: flexion and extension of the fingers (10 min), thumb opposition (10 min), adduction and abduction of the fingers (10 min), global movement of the hand (drinking water; 10 min)	5 days per week for additional intervention, 40 min	6 weeks	Baseline and postintervention	Upper extremity function MI^h^ NHPT^i^ Grip and pinch strength Quick-DASH^j^ Pain VAS^k^
Villafañe et al [29]	Standard rehabilitation (PT/OT) 5 days per week plus 30-min sessions for 3 days per week using the Gloreha program including number, fist, pinch, and synchronous.	Standard rehabilitation (PT/OT) 5 days per week plus 30-min sessions for 3 days per week using assisted stretching, shoulder, and arm exercises, and functional reaching tasks.	3 days per week for additional intervention, 30 min	3 weeks	Baseline and postintervention	Upper extremity function MI Quick-DASH Pain VAS ADL MBI Severity NHSS^l^

a ROM: range of motion.

b OT: occupational therapy.

c FMA-UE: The Fugl-Meyer Assessment for Upper Extremity.

d BBT: Box and Block Test.

e ADL: activity of daily living.

f MBI: Modified Barthel Index.

g PT: physical therapy.

h MI: Motricity Index.

i NHPT: Nine-Hole Peg Test.

j Quick-DASH: Quick Disabilities of the Arm, Shoulder, and Hand.

k VAS: visual analog scale.

l NIHSS: National Institutes of Health Stroke Scale.

### Comparator

All included trials used a conventional treatment as a comparator. Specifically, the activities reported included stretching of the affected arm and hand, passive hand movement, and reach-grasp-and-release activities. On average, the duration of the treatment was 30‐60 minutes per day, 2‐5 times per week, over a total period of 3‐6 weeks. In all trials, both the intensity and frequency of the rehabilitation programs were comparable between the study group and the control group.

### Outcome

#### Effect on Upper Extremity Function

Motor function: Two trials reported a significant difference between the baseline and end scores of the Motricity Index in the Gloreha training group [28,29]. One reported a significant difference between Gloreha training and conventional rehabilitation (*P*=.002) [28], while 1 trial reported a large effect size of Gloreha training (Cohen *d*=3.65) [29]. Lee et al [27] investigated the effect of Gloreha training on the upper extremity motor function through the Fugl-Mayer Assessment for upper extremity. In this study, the effect of Gloreha training was shown in the Fugl-Meyer Assessment for upper extremity proximal domain (*P*=.03) and the total score (*P*=.046) within the group.

Grip strength: A study by Vanoglio et al [28] shows a greater improvement in grip and pinch strength compared with conventional rehabilitation (*P*=.003 and *P*=.04). On the other hand, Lee et al [27] found no significant difference in grip strength from baseline (*P*=.55) and between groups (*P*=.59).

Dexterity: Vanoglio et al [28] measured the effect of Gloreha training on manual dexterity with the Nine-Hole Peg Test, and it showed a significant improvement compared with conventional rehabilitation (*P*=.009). Lee et al [27] used the Box and Block Test and found no statistical difference within and between groups (*P*=.10 and *P*=.79).

#### Functional Ability

The Quick Disabilities of the Arm, Shoulder, and Hand (Quick-DASH) was used to assess the functional ability of the upper extremity in 2 trials. Vanoglio et al [28] showed a significant difference in the Quick-DASH score between the Gloreha training group and conventional rehabilitation (*P*=.048). Another trial by Villafañe et al [29] found a significant improvement within the group. They also reported a small effect size of Gloreha training (Cohen *d*=0.42).

#### ADL Performance

In total, 2 trials investigated the effect of Gloreha training on ADL performance. Villafañe et al [29] found that the Barthel Index (BI) score for both groups was significantly improved from baseline with a moderate effect size (Cohen *d*=0.5). Lee et al [27] used a modified BI to measure the ADL performance and found that the Gloreha training showed a significant improvement from baseline (*P*=.04) but no significant difference between groups.

### Risk of Bias in Individual Studies

**Figure 2.**
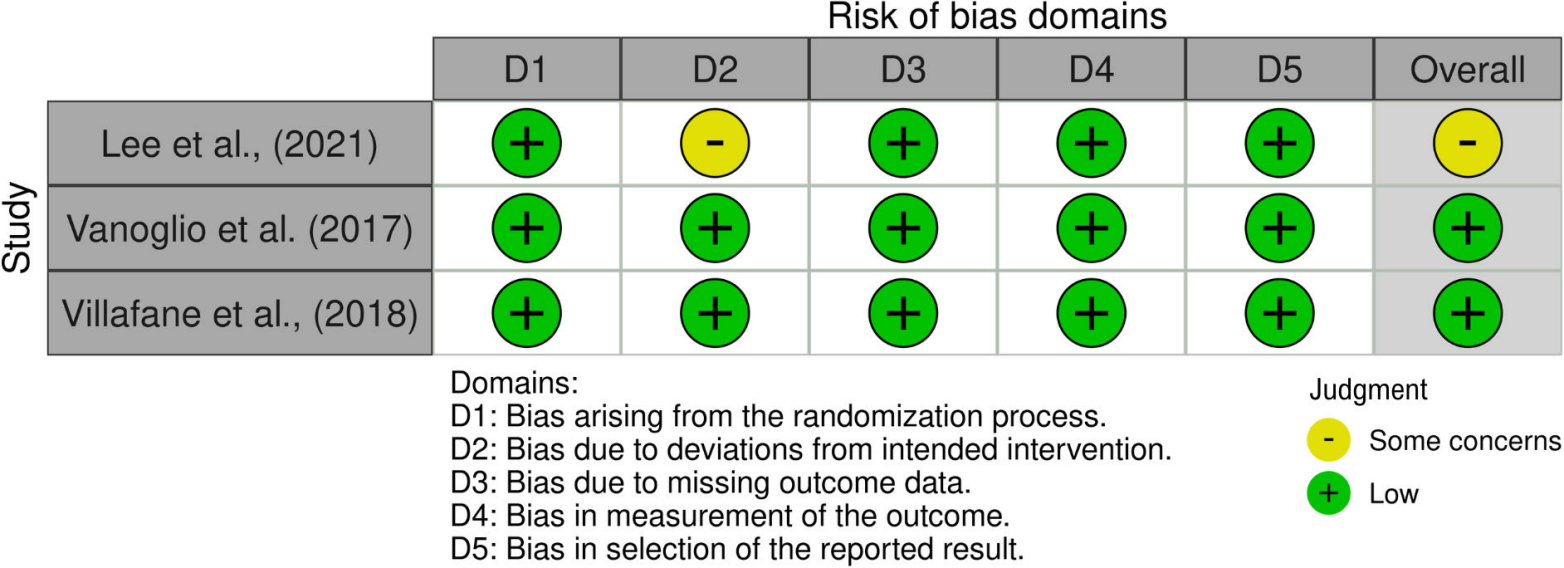
Risk of bias assessment [27-29].

Figure 2 illustrates the assessments of the risk of bias for individual studies. Overall, 2 out of 3 trials were deemed to be at low risk of bias [28,29]. However, 1 trial was judged as having some concerns regarding bias due to deviations from the intended intervention. This concern arose because it could be assumed that the reasons for participant dropout were related to the intervention context [27].

## Discussion

### Principal Results

This systematic review aimed to synthesize evidence on the effects of robot-assisted training using the Gloreha device on upper extremity function and ADLs in patients post stroke. Overall, the results suggest that Gloreha training, whether integrated with conventional rehabilitation or used independently, has the potential to enhance motor function and functional ability in survivors of stroke. However, multiple methodological issues, including risk of bias, heterogeneous interventions, and limited sensitivity of certain outcome measures, necessitate a more cautious and critical interpretation of these findings.

The evidence suggests that Gloreha training contributes to improvements in motor function, as demonstrated by the significant gains in the Motricity Index in 2 of the included trials [28,29]. The device appears to target upper extremity strength, with 1 study reporting a large effect size of Gloreha on motor recovery [29]. In addition, the improvements in the Fugl-Mayer Assessment scores, particularly in the proximal domain (which measures shoulder and elbow function), suggest that Gloreha training may be particularly beneficial for enhancing motor function [27]. The positive changes in the total score further support the overall effectiveness of the intervention in improving upper extremity motor function. The device’s design—combining passive movements with active-assisted and game modes [19]—might be a key contributor to these improvements, particularly in survivors of stroke who struggle with initiating movement.

Strength improvements were notable in one of the studies, with greater gains in grip and pinch strength in the Gloreha group compared with CT [28]. This aligns with existing literature on RAT, where repetitive, high-intensity practice has been shown to enhance motor recovery [30-32]. The Gloreha device provides consistent and controlled finger and hand movements [19], which are likely critical for rebuilding strength in the affected limbs. However, one study did not observe significant improvements in grip strength [27]. This discrepancy may stem from differences in participant characteristics, particularly in stroke chronicity. Vanoglio et al [28] included individuals with stroke in the acute to subacute phase (less than 3 mo post stroke), whereas Lee et al [27] enrolled participants in the chronic phase (more than 6 mo post stroke) as defined by Bernhardt et al [33]. Since spontaneous neurological recovery is most pronounced in the early poststroke period, reaching a plateau around 3 months and slowing thereafter [34], the diminished neuroplastic potential in chronic-phase patients could contribute to the less favorable outcomes observed in grip strength improvements.

Functional ability, assessed through the Quick-DASH, also showed improvements in some studies. For example, significant differences were observed between the Gloreha group and CT in 1 trial [28]. These functional ability improvements could potentially impact patients’ daily lives, as the Quick-DASH assessment is designed to measure symptoms and ability to perform various activities, which are particularly relevant for survivors of stroke who often experience upper extremity impairments [35,36]. These findings indicate that the Gloreha device may have a valuable role in enhancing upper extremity function and functional capability.

The observed improvements in motor function and strength align with existing studies on robotic-assisted rehabilitation, which have shown that RAT could promote motor recovery and functional improvements in survivors of stroke [17,20,21]. The repetitive, high-intensity practice provided by the Gloreha device facilitates motor relearning by enhancing cortical plasticity [16,37,38]. Robotic devices like Gloreha offer the advantage of delivering precise, task-specific movements crucial for retraining motor circuits [39].

An added feature of Gloreha is its action observation component, which allows patients to observe hand movements on a screen before attempting them [19]. While this mechanism may theoretically stimulate neural pathways involved in motor learning [40], empirical validation within the included studies was limited. Caution is warranted in generalizing potential benefits from action observation without stronger direct evidence. This consideration aligns with experience-dependent plasticity theories [37,38], which posit that repeated, meaningful practice reinforces neural connections in the damaged motor cortex.

While Gloreha demonstrated significant improvements in motor function and strength, its effect on ADL performance was less pronounced. Furthermore, 2 of the studies found improvements in ADL performance within groups, but no significant differences between Gloreha and conventional rehabilitation were observed [27,29]. This finding is consistent with previous meta-analyses, which have shown that while RAT can improve upper extremity function, it often does not translate into significant enhancements in ADLs [17,41]. Potential explanations include the low sensitivity of certain ADL scales, insufficient intensity or duration of the Gloreha training, and a lack of comprehensive, task-oriented practice that might better transfer to real-world functional tasks. The BI, used to assess ADL performance in these trials, may lack the sensitivity needed to detect subtle changes in patients, especially those with mild or severe impairments [42,43]. More sensitive outcome measures, such as the modified Rankin Scale [42] or functional independence measure [43], could provide more accurate assessments of functional gains. Also, the dosage of Gloreha training in these studies was less than 15 hours, below the threshold identified in previous research for translating motor improvements into significant functional gains [44]. Higher intensity and longer training durations might be necessary to induce clinically meaningful changes in ADL performance. Furthermore, research highlighted that high-intensity training alone may be inadequate if the quality of tasks is not sufficiently relevant to daily living [44].

Several factors may account for the variability in outcomes across the included trials. First, the intervention protocols differed substantially—ranging from passive to active-assisted modes, with varying frequencies and session lengths—which may obscure the dose-response relationship and hamper replication. Second, risk of bias remains a concern as the participant dropout in Lee et al [27] could have introduced attrition bias, and small sample sizes in other trials reduce the generalizability of findings. Third, the inconsistency in stroke phase (acute versus chronic) among participants raises questions about whether Gloreha’s beneficial effects might diminish over time or require different protocols for long-term recovery. Earlier work [45] showed no superiority for fully robot-driven movements compared with CT, underscoring the value of active engagement. Indeed, active-assisted modes that require patient effort appear to yield more robust outcomes [45,46].

Furthermore, neurorehabilitation principles emphasize the importance of task-specific practice for motor learning and brain plasticity [38,47]. Task-oriented approaches, which integrate real-world tasks into therapy, have been shown to improve ADL performance in patients with stroke [48]. It is possible that integrating Gloreha into a more comprehensive, task-oriented rehabilitation program could yield better functional outcomes.

For clinicians, these findings underscore the potential of the Gloreha device to improve upper extremity strength and function in patients with stroke. It may be particularly useful for individuals in the acute, subacute, or chronic phases of recovery. The combination of passive and active-assisted modes allows the device to be adapted to patients with varying levels of impairment. However, the variability in outcomes suggests that Gloreha is not a one-size-fits-all solution. Clinicians should assess each patient’s specific needs and consider combining Gloreha training with other interventions, such as task-oriented training, to maximize recovery.

Further research is needed to optimize the use of Gloreha in stroke rehabilitation, particularly regarding the ideal dosage, timing, and integration with other therapies. In addition, long-term studies are required to determine the sustainability of motor and functional improvements. Finally, comparing the clinical and cost-effectiveness of Gloreha with other robotic devices will be crucial for informing health care policies and reimbursement decisions.

### Limitation

While the examined evidence base provides valuable insights, it is important to acknowledge certain limitations. First, the number of included studies was relatively small (n=3), with a total sample size of only 83 participants across the trials. This limited sample may affect the precision and generalizability of the findings. However, considering the comprehensive search employed in this study, we are certain that our findings represent the existing evidence in the field. In addition, the studies varied in terms of time since stroke onset, with some focused on the acute or subacute phase and one on the chronic phase. Rehabilitation effects may differ based on stroke chronicity. Furthermore, the trials had somewhat heterogeneous intervention protocols in terms of Gloreha modes used, duration or frequency of training sessions, and comparator treatments.

### Conclusions

This systematic review revealed that the Gloreha robotic device shows promise for improving motor function in patients post stroke, particularly in upper extremity strength and dexterity. However, its impact on ADLs remains inconsistent, possibly due to limited task specificity in training, insensitive outcome measures, and insufficient training dosage, as most studies provided less than 15 hours of intervention—below the threshold suggested for meaningful functional gains. Future research should explore integrating Gloreha with task-oriented rehabilitation, assess its long-term effects on specific ADL tasks, and include a broader range of survivors of stroke to determine its optimal dosage and functional utility. Clinicians should use Gloreha as a motor recovery tool but combine it with other interventions to enhance real-world functional outcomes.

## Supplementary material

10.2196/68268 Multimedia Appendix 1 Keywords used in searching strategies.

10.2196/68268 Multimedia Appendix 2 Characteristics of excluded studies.

10.2196/68268 Checklist 1 PRISMA (Preferred Reporting Items for Systematic Reviews and Meta-Analyses) checklist.

## Abbreviations

ADL: activity of daily living
BI: Barthel Index
CT: conventional therapy
PRISMA: Preferred Reporting Items for Systematic Reviews and Meta-Analyses
Quick-DASH: Quick Disabilities of the Arm, Shoulder, and Hand
RAT: robot-assisted therapy
RCT: randomized controlled trial
RoB 2: Revised Cochrane risk-of-bias tool for randomized trials
