# Supplementary material for: Effectiveness of Robot-Assisted Upper Extremity Function Training (Gloreha) on Upper Extremities Function After Stroke: Systematic Review
Source: JMIR Rehabil Assist Technol. 2025 Jun 5;12:e68268. doi: 10.2196/68268 (PMC12162109; doi:10.2196/68268)
Supplement: Multimedia Appendix 1 [file rehab-v12-e68268-s001.docx]

Appendix1: Keywords used in searching strategies

| Database | Search strategy |
| --- | --- |
| Pubmed | ("Stroke rehabilitation") AND (("stroke") OR ("subacute stroke") OR ("sub-acute stroke") OR ("CVA") OR ("cerebrovascular accident") OR ("cerebral stroke")) AND (("upper limb") OR ("upper extremity") OR ("arm") OR ("hand") OR ("wrist")) AND (("Gloreha") OR (“robotic glove”) OR (“Robot-assisted therapy”) OR (“Robot*-assisted ”) OR (“RAT”) OR (“robotic device”)) AND (("rehabilitation") OR ("training") OR ("therapy") OR ("program") OR ("device")) AND (("usual care") OR ("treatment-as-usual") OR ("rehabilitation") OR ("upper-limb train*") OR ("standard care") OR ("Conventional rehabilitation") OR ("Physiotherapy") OR ("physical therapy") OR ("occupational therapy")) AND (("function") OR ("impairment") OR ("disability") OR ("movement") OR ("strength") OR ("activity") OR ("ADL")) |
| Embase | ('stroke rehabilitation'/exp OR 'stroke rehabilitation') AND ('stroke'/exp OR 'stroke' OR 'subacute stroke'/exp OR 'subacute stroke' OR 'sub-acute stroke' OR 'cva'/exp OR 'cva' OR 'cerebrovascular accident'/exp OR 'cerebrovascular accident' OR 'cerebral stroke'/exp OR 'cerebral stroke') AND ('upper limb'/exp OR 'upper limb' OR 'upper extremity'/exp OR 'upper extremity' OR 'arm'/exp OR 'arm' OR 'hand'/exp OR 'hand' OR 'wrist'/exp OR 'wrist') AND ('gloreha' OR 'robotic glove' OR 'robot-assisted therapy' OR 'robot*-assisted' OR 'rat'/exp OR 'rat' OR 'robotic device'/exp OR 'robotic device') AND ('rehabilitation'/exp OR 'rehabilitation' OR 'training'/exp OR 'training' OR 'therapy'/exp OR 'therapy' OR 'program'/exp OR 'program' OR 'device'/exp OR 'device') AND ('usual care'/exp OR 'usual care' OR 'treatment-as-usual' OR 'rehabilitation'/exp OR 'rehabilitation' OR 'upper-limb train*' OR 'standard care'/exp OR 'standard care' OR 'conventional rehabilitation' OR 'physiotherapy'/exp OR 'physiotherapy' OR 'physical therapy'/exp OR 'physical therapy' OR 'occupational therapy'/exp OR 'occupational therapy') AND ('function'/exp OR 'function' OR 'impairment'/exp OR 'impairment' OR 'disability'/exp OR 'disability' OR 'movement'/exp OR 'movement' OR 'strength'/exp OR 'strength' OR 'activity'/exp OR 'activity' OR 'adl') |
| CINAHL | ("Stroke rehabilitation") AND (("stroke") OR ("subacute stroke") OR ("sub-acute stroke") OR ("CVA") OR ("cerebrovascular accident") OR ("cerebral stroke")) AND (("upper limb") OR ("upper extremity") OR ("arm") OR ("hand") OR ("wrist")) AND (("Gloreha") OR (“robotic glove”) OR (“Robot-assisted therapy”) OR (“Robot*-assisted ”) OR (“RAT”) OR (“robotic device”)) AND (("rehabilitation") OR ("training") OR ("therapy") OR ("program") OR ("device")) AND (("usual care") OR ("treatment-as-usual") OR ("rehabilitation") OR ("upper-limb train*") OR ("standard care") OR ("Conventional rehabilitation") OR ("Physiotherapy") OR ("physical therapy") OR ("occupational therapy")) AND (("function") OR ("impairment") OR ("disability") OR ("movement") OR ("strength") OR ("activity") OR ("ADL")) |
| EBSCO Open Dissertations | ("Stroke rehabilitation") AND (("stroke") OR ("subacute stroke") OR ("sub-acute stroke") OR ("CVA") OR ("cerebrovascular accident") OR ("cerebral stroke")) AND (("upper limb") OR ("upper extremity") OR ("arm") OR ("hand") OR ("wrist")) AND (("Gloreha") OR (“robotic glove”) OR (“Robot-assisted therapy”) OR (“Robot*-assisted ”) OR (“RAT”) OR (“robotic device”)) AND (("rehabilitation") OR ("training") OR ("therapy") OR ("program") OR ("device")) AND (("usual care") OR ("treatment-as-usual") OR ("rehabilitation") OR ("upper-limb train*") OR ("standard care") OR ("Conventional rehabilitation") OR ("Physiotherapy") OR ("physical therapy") OR ("occupational therapy")) AND (("function") OR ("impairment") OR ("disability") OR ("movement") OR ("strength") OR ("activity") OR ("ADL")) |
| The Cochrane Central Register  of Controlled Trials | ("Stroke rehabilitation") AND (("stroke") OR ("subacute stroke") OR ("sub-acute stroke") OR ("CVA") OR ("cerebrovascular accident") OR ("cerebral stroke")) AND (("upper limb") OR ("upper extremity") OR ("arm") OR ("hand") OR ("wrist")) AND (("Gloreha") OR ("robotic glove") OR ("Robot-assisted therapy") OR ("Robot*-assisted") OR ("RAT") OR ("robotic device")) AND (("rehabilitation") OR ("training") OR ("therapy") OR ("program") OR ("device")) AND (("usual care") OR ("treatment-as-usual") OR ("rehabilitation") OR ("upper-limb train*") OR ("standard care") OR ("Conventional rehabilitation") OR ("Physiotherapy") OR ("physical therapy") OR ("occupational therapy")) AND (("function") OR ("impairment") OR ("disability") OR ("movement") OR ("strength") OR ("activity") OR ("ADL")) |

Appendix 2: Characteristics of excluded studies.

| Reason for exclusion | Studies |
| --- | --- |
| Not RCT | 1. Crema A, et al. Neuromuscular electrical stimulation restores upper limb sensory-motor functions and body representations in chronic stroke survivors. Med. 2022 Jan 14;3(1):58-74.e10. |
|  | 2. Giulia M, Francesca M, Simone T, Barbara V, Erica P, Francesco L. Is passive mobilization robot-assisted therapy effective in upper limb motor recovery in patients with acquired brain injury? A randomized crossover trial. Int. j. phys. ther. rehabil. 2016 Apr 4;2(1). |
|  | 3. Borboni A, et al. Robot-assisted rehabilitation of hand paralysis after stroke reduces wrist edema and pain: A prospective clinical trial. J Manipulative Physiol Ther. 2017 Jan;40(1):21-30. |
