# Supplementary material for: Effectiveness of Robot-Assisted Upper Extremity Function Training (Gloreha) on Upper Extremities Function After Stroke: Systematic Review
Source: JMIR Rehabil Assist Technol. 2025 Jun 5;12:e68268. doi: 10.2196/68268 (PMC12162109; doi:10.2196/68268)
Supplement: Multimedia Appendix 2 [file rehab-v12-e68268-s002.docx]

Appendix 2: Characteristics of excluded studies.

| Reason for exclusion | Studies |
| --- | --- |
| Not RCT | 1. Crema A, et al. Neuromuscular electrical stimulation restores upper limb sensory-motor functions and body representations in chronic stroke survivors. Med. 2022 Jan 14;3(1):58-74.e10. |
|  | 2. Giulia M, Francesca M, Simone T, Barbara V, Erica P, Francesco L. Is passive mobilization robot-assisted therapy effective in upper limb motor recovery in patients with acquired brain injury? A randomized crossover trial. Int. j. phys. ther. rehabil. 2016 Apr 4;2(1). |
|  | 3. Borboni A, et al. Robot-assisted rehabilitation of hand paralysis after stroke reduces wrist edema and pain: A prospective clinical trial. J Manipulative Physiol Ther. 2017 Jan;40(1):21-30. |
